# Analysis of the PD-1 Ligands Among Gastrointestinal Cancer Patients: Focus on Cancer Immunity

**DOI:** 10.3389/fonc.2021.637015

**Published:** 2021-03-23

**Authors:** Lin Dai, Zilin Huang, Wang Li

**Affiliations:** ^1^Cancer Prevention Center, Sun Yat-sen University Cancer Center, Guangzhou, China; ^2^State Key Laboratory of Oncology in South China, Guangzhou, China; ^3^Collaborative Innovation Center for Cancer Medicine, Guangzhou, China; ^4^Department of Medical Imaging and Interventional Radiology, Sun Yat-sen University Cancer Center, Guangzhou, China

**Keywords:** gastrointestinal cancer, CD274, PDCD1LG2, prognosis, cancer immunity

## Abstract

Many types of gastrointestinal cancer have shown promising outcomes after checkpoint blockade immunotherapy; however, it remains largely unclear about the expression profiles of programmed death 1 (PD-1) ligands (CD274 and PDCD1LG2) in the context of human pan-cancer. This work comprehensively analyzed the expression pattern of the PD-1 ligands and the clinical significance in the prognosis prediction among the seven types of gastrointestinal malignancies collected from The Cancer Genome Atlas (TCGA) and the Cancer Cell Line Encyclopedia (CCLE) database. Furthermore, the correlation of CD274/PDCD1LG2 with cancer immunity was also explored. The patients with liver hepatocellular carcinoma (LIHC) receiving cytokine-induced killer (CIK) cell immunotherapy at our cancer center were enrolled. CD274 and PDCD1LG2 displayed inconsistent gene expression levels among the diverse cancer cell lines. Typically, the abnormal expression level of CD274 and PDCD1LG2 was detected in both esophageal carcinoma (ESCA) and stomach adenocarcinoma (STAD), where PDCD1LG2 was related to the overall survival (OS) of the patients in ESCA (*p* = 0.015) and STAD (*p* = 0.025). High-serum CD274 and PDCD1LG2 levels predicted a worse survival in the patients with LIHC receiving CIK therapy. More importantly, the expression level of CD274 and PDCD1LG2 was significantly correlated with the degree of Estimation of STromal and Immune cells in MAlignant Tumor tissues using the Expression data (ESTIMATE). In addition, we found that CD274 and PDCD1LG2 were correlated with gene markers in tumor-infiltrating immune cells. Furthermore, the expression of CD274 and PDCD1LG2 was correlated with tumor mutation burden (TMB), microsatellite instability (MSI), mismatch repair (MMR), and DNA methyltransferase (DNMT) of different types of cancers. The present work comprehensively analyzed a RNA sequencing of the PD-1 ligands across the seven distinct types of gastrointestinal cancers, which provided clues for further studies in cancer immunity and development.

## Introduction

Gastrointestinal malignant tumor is a leading cause of death (1). The development of immunotherapy using immune checkpoint inhibitors has achieved breakthroughs in treating cancers. Programmed death 1 (PD-1) and its ligands, programmed death ligand 1 (PD-L1, or CD274) and PD-L2 (PDCD1LG2), are known as an immune checkpoint axis available to the cancer cells escaping from the immune destruction of T cells (2). In other words, PD-1 binds two ligands CD274 and PDCD1LG2 to inhibit the T-cell activation and invalidate immune surveillance. The results from experimental investigations and clinical studies showed that PD-1/PD-L1 blockers were demonstrated to be effective in gastrointestinal cancers (3, 4). However, a specific gene expression of CD274/PDCD1LG2 in gastrointestinal cancers at a pan-cancer level remains largely unknown.

A pan-cancer analysis is initially utilized in the field of cancer research to shed more light on the common features and heterogeneities of various human malignancies (5). A pan-cancer analysis, which is also referred to as an analysis of molecular abnormalities among several cancer types, is able to identify the common features and heterogeneities of some vital dysregulated biological processes in diverse cancer cell lineages. The project of a pan-cancer analysis, including Cancer Cell Line Encyclopedia (CCLE) and The Cancer Genome Atlas (TCGA), has been established based on the different human cancer cell lines and tissues at epigenomic, genomic, proteomic, and transcriptomic levels (6–8). Recently, a pan-cancer analysis is adopted to identify certain functional and pathway genes, which allows to specifically, comprehensively, and thoroughly understand human cancers. Considering as an example, the tumor hypoxia-associated multi-omic molecular characteristics were investigated, and some molecular alterations were suggested to be correlated with the sensitivity or resistance to antitumor agents. It contributed to a comprehensive understanding of tumor hypoxia at the molecular level and has certain implications for cancer treatment in clinical practice (9). The updated data regarding the frequency, etiology, and outcomes of the forkhead box protein M1 (FOXM1) upregulation in human cancers were defined among 33 cancer types derived from TCGA databases (10). Notably, the information obtained from these 33 TCGA-derived cancers had systemically revealed the long non-coding RNA- (lncRNA-) mediated dysregulation in cancers, which also provides the precious approach and resources to investigate the lncRNAs functions in cancers (11). It is beneficial to characterize the frequency of occurrence and variability of immune phenotypes within and among various cancer types, so as to understand the immune status for those untreated cancers. Typically, this method has been applied in more than 9,000 expression data of TCGA-derived cancer genes (12). Therefore, a pan-cancer analysis helps to illustrate the patterns that are beneficial for developing the combination treatments and individualized therapies.

In this regard, taking advantage of the large data sets from TCGA, this study aimed to examine the expression profiles of CD274/PDCD1LG2 and the prognostic significance in human gastrointestinal tumors. In addition, the association between CD274/PDCD1LG2 and the tumor infiltration level, tumor mutation burden (TMB), microsatellite instability (MSI), mismatch repair (MMR), and DNA methyltransferase (DNMT) were analyzed in the different tumor types. Our study helps to understand the vital roles of CD274/PDCD1LG2 in gastrointestinal tumor-immune interactions.

## Materials and Methods

### Patient Data Sets and Processing

The Cancer Genome Atlas, a milestone of the cancer genomics project, characterizes thousands of primary cancer samples and matched the characterized samples with the adjacent non-carcinoma samples from seven types of cancers, including cholangiocarcinoma (CHOL, *N* = 45), colon adenocarcinoma (COAD, *N* = 521), esophageal carcinoma (ESCA, *N* = 173), liver hepatocellular carcinoma (LIHC, *N* = 424), pancreatic adenocarcinoma (PAAD, *N* = 182), rectum adenocarcinoma (READ, *N* = 177), and stomach adenocarcinoma (STAD, *N* = 407). In this study, the TCGA level three RNA sequencing processed data and the corresponding clinical annotations were acquired by using the University of California Santa Cruz (UCSC) cancer genome browser (https://tcga.xenahubs.net, accessed April 2020). The gene expression data of healthy samples from the Genotype-Tissue Expression (GTEx) database (https://gtexportal.org/home/) was also downloaded for a comparison. The CCLE public project is established through a comprehensive characterization of tremendous human tumor models at both genetic and pharmacological levels. To examine the differential gene expression in cancers at a larger scale, the CCLE database containing the RNA-sequencing data sets for over 1,000 cell lines (https://portals.broadinstitute.org/ccle) was used in this study. Meanwhile, the approval from the Ethics Committee was exempted since only the open-access data were used. A total of 122 consecutive patients with LIHC receiving an adjuvant cytokine-induced killer (CIK) cell immunotherapy after a curative resection at our center were retrospectively enrolled, which were approved by the Ethics Committee of Sun Yat-sen University Cancer Center. A detailed treatment workflow is described in our previous study (13).

### Gene Expression and Survival Analysis

To compare the gene expression levels between cancer and adjacent non-carcinoma samples, the data regarding the gene expression profiles of CD274 and PDCD1LG2 were extracted from the seven cancer types in TCGA to form an expression matrix. Thereafter, the formed expression matrix and clinical information were matched with a patient ID. Receiver operating characteristic (ROC) curve was drawn to evaluate the ability of CD274 and PDCD1LG2 to distinguish the tumor sample from the normal sample. Moreover, the Kaplan–Meier (KM) analysis was conducted to compare the overall survival (OS) of the patients with TCGA cancer stratified based on the gene expression level of CD274 and PDCD1LG2 using a log-rank test.

### PD-1 Ligands and Tumor Immunity

The Estimation of STromal and Immune cells in MAlignant Tumor tissues using the Expression data (ESTIMATE) is an approach that uses gene expression profiles to predict the tumor purity and the infiltrating stromal cells/immunocytes in tumor tissues (14). The ESTIMATE algorithm produces three scores based on the gene set enrichment analysis (GSEA) of a single sample, including (1) stromal score, which determines stromal cells in tumor tissues, (2) immune score, which stands for the immunocyte infiltration level in tumor tissues, and (3) estimate score, which infers the tumor purity. In this study, we used the ESTIMATE algorithm to estimate the immune and stromal scores in tumor tissues according to the corresponding transcriptional data. Later, we calculated the correlation of these scores with the expression of CD274 and PDCD1LG2.

Additionally, we examined the association of the expression level of CD274 and PDCD1LG2 with the gene markers in the selected tumor-infiltrating immunocytes with reference to the previous research studies (15–17). The estimated statistical significance and the Spearman's correlation coefficient were generated through a correlation analysis. Thereafter, we plotted an expression heatmap between a pair of genes in a specific type of cancer.

Tumor mutation burden measures the number of mutations in a specific cancer genome. Numerous articles have been conducted to explore the significance of using the TMB status as a biomarker to predict the patients with the highest response to checkpoint inhibitors (18). In this study, the somatic mutation data of all patients with TCGA were downloaded (https://tcga.xenahubs.net), their TMB scores were calculated, and the correlation between TMB and CD274/PDCD1LG2 was determined. MSI is characterized by the widespread length polymorphisms of microsatellite sequences resulting from DNA polymerase slippage. Recently, it was suggested that the patients with high MSI cancers can gain benefits from immunotherapy, and MSI was used as the genetic instability index for cancer detection (19). In this study, we calculated the MSI score for each patient, followed by a correlation analysis between MSI and CD274/PDCD1LG2. Notably, MMR, also referred to as the normal tissue DNA repair system, can correct errors in the process of DNA replication. However, due to the lack of MMR genes in tumor cells or the defects during the replication repair, the possibility of gene mutation has increased (19). Later, we performed a correlation analysis between MMR genes (including MLH1, MSH2, MSH6, PMS2, and EPCAM) and CD274/PDCD1LG2. DNMT participates in the process of tumorigenesis and development, whereas DNMT1, DNMT3A, and DNMT3B are the major key enzymes that catalyze DNMT (20). We also analyzed a correlation between DNMTs and CD274/PDCD1LG2.

### Statistical Analysis

In the present work, the clinical survival types, including OS and progression-free interval (PFI), were selected for analysis. Generally, OS is deemed as the duration from the date of diagnosis to the date of death due to any cause, while PFI is defined as the disease progression or death from any cause.

The Wilcox log-rank test was adopted to determine the presence or absence of a markedly increased sum of the gene expression z-scores in cancer tissues compared with the adjacent normal tissues. The area under the ROC curve was calculated to evaluate the ability of CD274 and PDCD1LG2 in distinguishing the tumor sample from the normal sample. Meanwhile, the Kruskal–Wallis test was employed to compare the difference in the expression of CD274 and PDCD1LG2 in cell lines. Survival was analyzed by the KM curves and a log-rank test. The Spearman test was utilized for a correlation analysis. The R language (version 3.6.0; R Foundation) was used for all analyses. A two-sided difference of *p* < 0.05 indicated statistical significance.

## Results

### Pan-Cancer Expression Landscape of CD274 and PDCD1LG2

**Figure 1:**
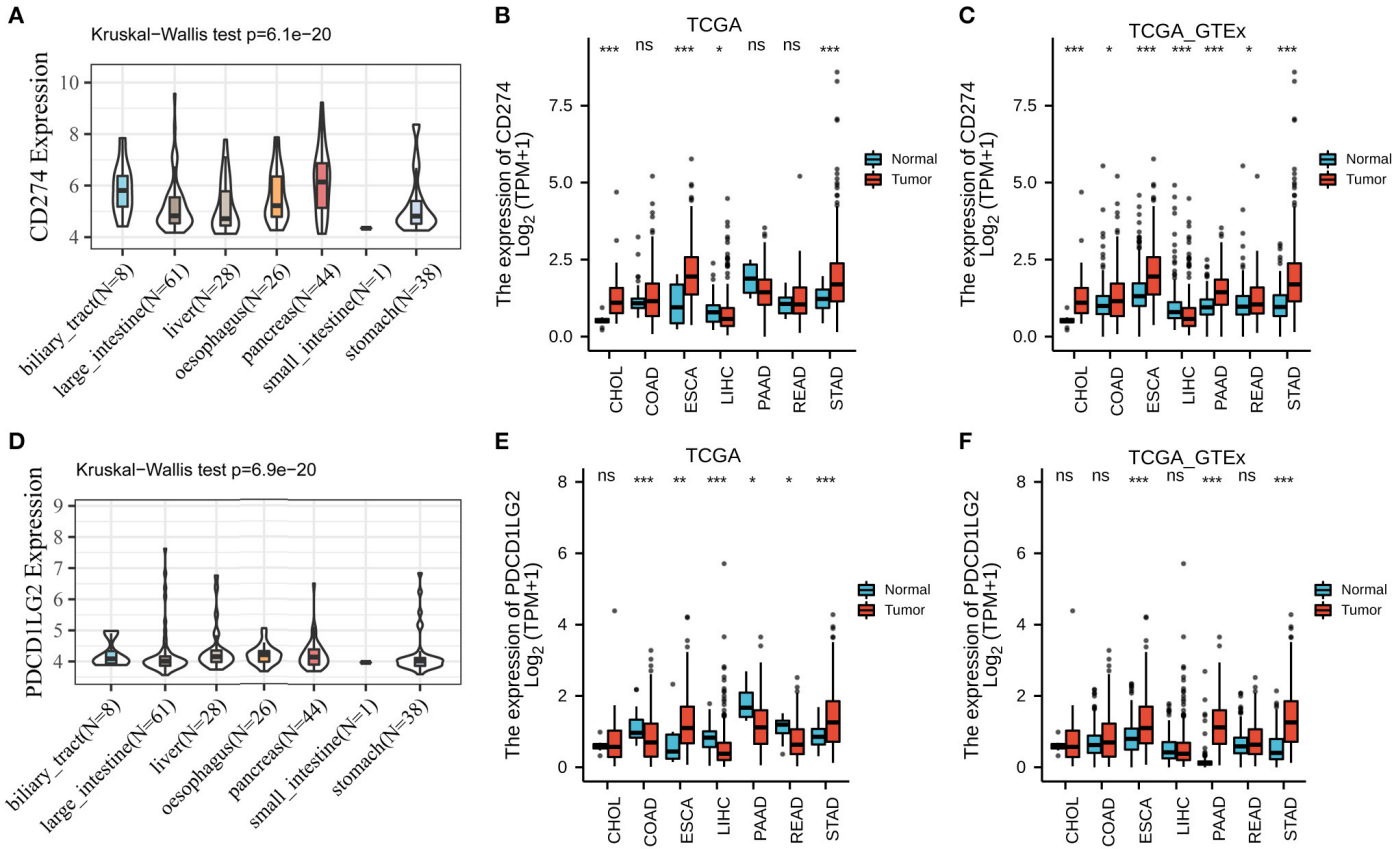
CD274/PDCD1LG2 expression level in human pan-cancer. **(A)** The messenger RNA (mRNA) level of CD274 in Cancer Cell Line Encyclopedia (CCLE). **(B)** The mRNA level of CD274 in The Cancer Genome Atlas (TCGA). **(C)** CD274 mRNA level in TCGA and genotype-tissue expression (GTEx). **(D)** The mRNA level of PDCD1LG2 in CCLE. **(E)** The mRNA level of PDCD1LG2 in TCGA. **(F)** PDCD1LG2 mRNA level in TCGA and GTEx. *p < 0.05; **p < 0.01; ***p < 0.001; ns, not significant.

**Figure 2:**
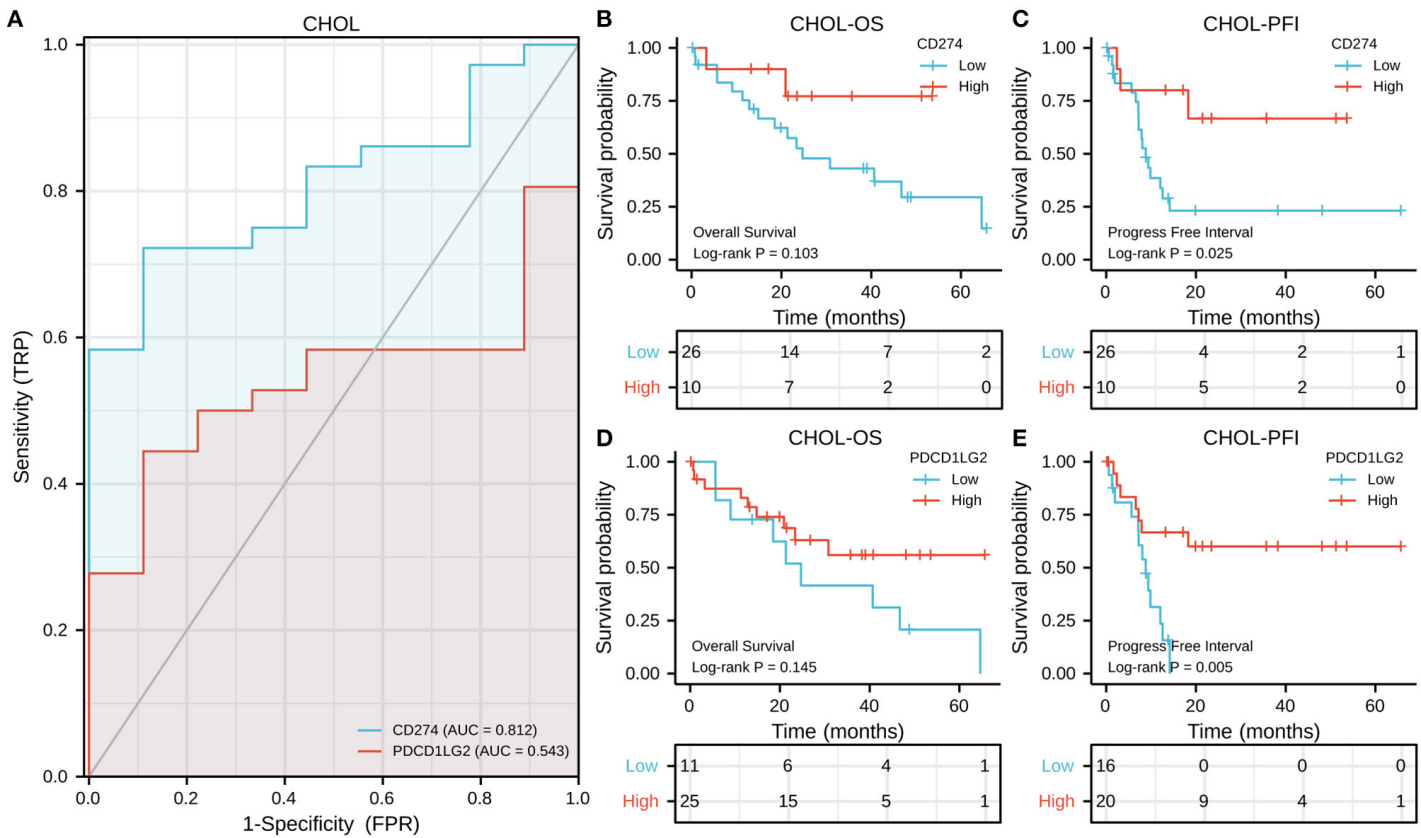
CD274/PDCD1LG2 expression and its correlation with cholangiocarcinoma (CHOL) patient survival. **(A)** Receiver operating characteristic (ROC) curves CD274/PDCD1LG2 of distinguishing tumor from normal samples. **(B,C)** The Kaplan–Meier (KM) analysis shows the association between the CD274/PDCD1LG2 expression and overall survival (OS). **(D,E)** CD274/PDCD1LG2 expression and progression-free interval (PFI).

According to the results of CCLE analysis, CD274 and PDCD1LG2 showed inconsistent gene expression levels among the diverse cancer cell lines (*p* = 6.1e-20 and 6.9e-20, Figures 1A,D), where pancreas cells had a relatively higher gene expression. Consistent with the different gene expression levels in CCLE, CD274, and PDCD1LG2 also displayed distinct expression in TCGA. For the seven TCGA-derived cancer types, we detected that ESCA, PAAD, and STAD showed a relatively higher gene expression. The expression landscapes of CD274 and PDCD1LG2 in TCGA cohorts are presented in Figures 1B,E, respectively. In addition, CD274 and PDCD1LG2 also exhibited a higher gene expression when more samples from GTEx were added Figures 1C,F. Figure 2A shows the AUC values of CD274 (0.812) and PDCD1LG2 (0.543) in the patients with CHOL, and the AUC results of the other six cancer types are presented in Supplementary Figures 1–6.

### Screening of the Survival-Associated Cancers

In the OS analysis, KM analysis showed that the subjects with high CD274 levels had short OS compared with those with low CD274 levels in PAAD (*p* = 0.012, Supplementary Figure 4B) whereas those with an increased CD274 expression showed superior OS to those with a decreased CD274 expression in COAD (*p* = 0.001, Supplementary Figure 1B); the subjects with a high PDCD1LG2 expression had poorer OS than those with a low PDCD1LG2 expression in STAD (*p* = 0.025, Supplementary Figure 6D) while the subjects with an increased PDCD1LG2 expression showed superior OS to those with a decreased PDCD1LG2 expression in ESCA (*p* = 0.015, Supplementary Figure 2D). In CHOL, no significant overall survival difference was seen between high and low gene expression patients (both *p* values of CD274 and PDCD1LG2 were more than 0.05, Figures 2B,D).

In the PFI analysis, according to the results of KM analysis, the subjects with a high CD274 expression had poor PFI relative to those with a low CD274 expression in PAAD (*p* = 0.027, Supplementary Figure 4C); whereas the subjects having an increased CD274 expression showed superior PFI than those having a decreased CD274 expression in CHOL (*p* = 0.025, Figure 2C), as shown in Supplementary Figure 3; high PDCD1LG2 predicted superior prognosis for CHOL (*p* = 0.005, Figure 2E) and LIHC (*p* = 0.003, Supplementary Figure 3E).

In our cancer center patients with LIHC, the median serum CD274 and PDCD1LG2 level were 5.1 and 14.7 μg/μl, respectively. When patients were classified using the best statistical cutoff values (CD274 = 11.22 μg/μl and PDCD1LG2 = 27.67 μg/μl), survival analysis showed that the patients with LIHC with a high level of CD274 and PDCD1LG2 had worse disease-free survival after CIK immunotherapy (both *p* < 0.01, Supplementary Figure 7).

### Expression of CD274/PDCD1LG2 Was Related to the Immune Infiltration Level

**Figure 3:**
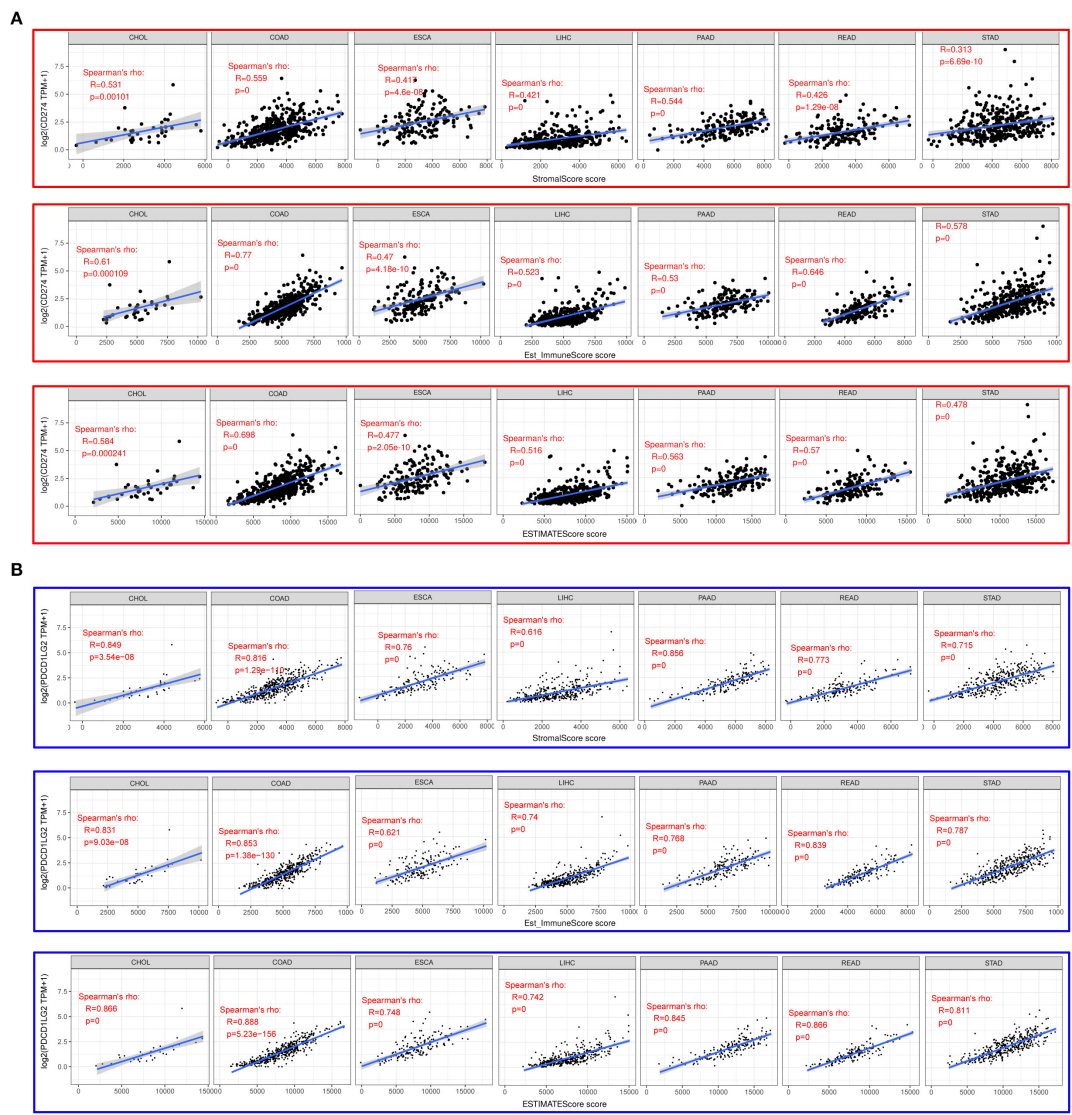
CD274/PDCD1LG2 expression is correlated with cancer immunity in Estimation of STromal and Immune cells in MAlignant Tumor tissues using the Expression data (ESTIMATE). **(A)** ESTIMATE predicting CD274 expression is correlated with tumor immune infiltration level across seven cancer types. Top panel, stromal score; middle panel, immune score; and bottom panel, estimate score. **(B)** ESTIMATE predicting PDCD1LG2 expression is correlated with tumor immune infiltration level across seven cancer types. Top panel, stromal score; middle panel, immune score; and bottom panel, estimate score.

The ESTIMATE method is developed to calculate the immune and stromal scores of cancer tissues. By adopting the ESTIMATE method, we calculated the immune, stromal, and estimate scores, respectively. Later, we evaluated the relationships between the immune/stromal scores and CD274/PDCD1LG2 expression. Figure 3 exhibits the results in these seven cancer types. Clearly, CD274/PDCD1LG2 expression was significantly correlated with the stromal, immune, and estimate scores (all values of *p* < 0.05).

**Figure 4:**
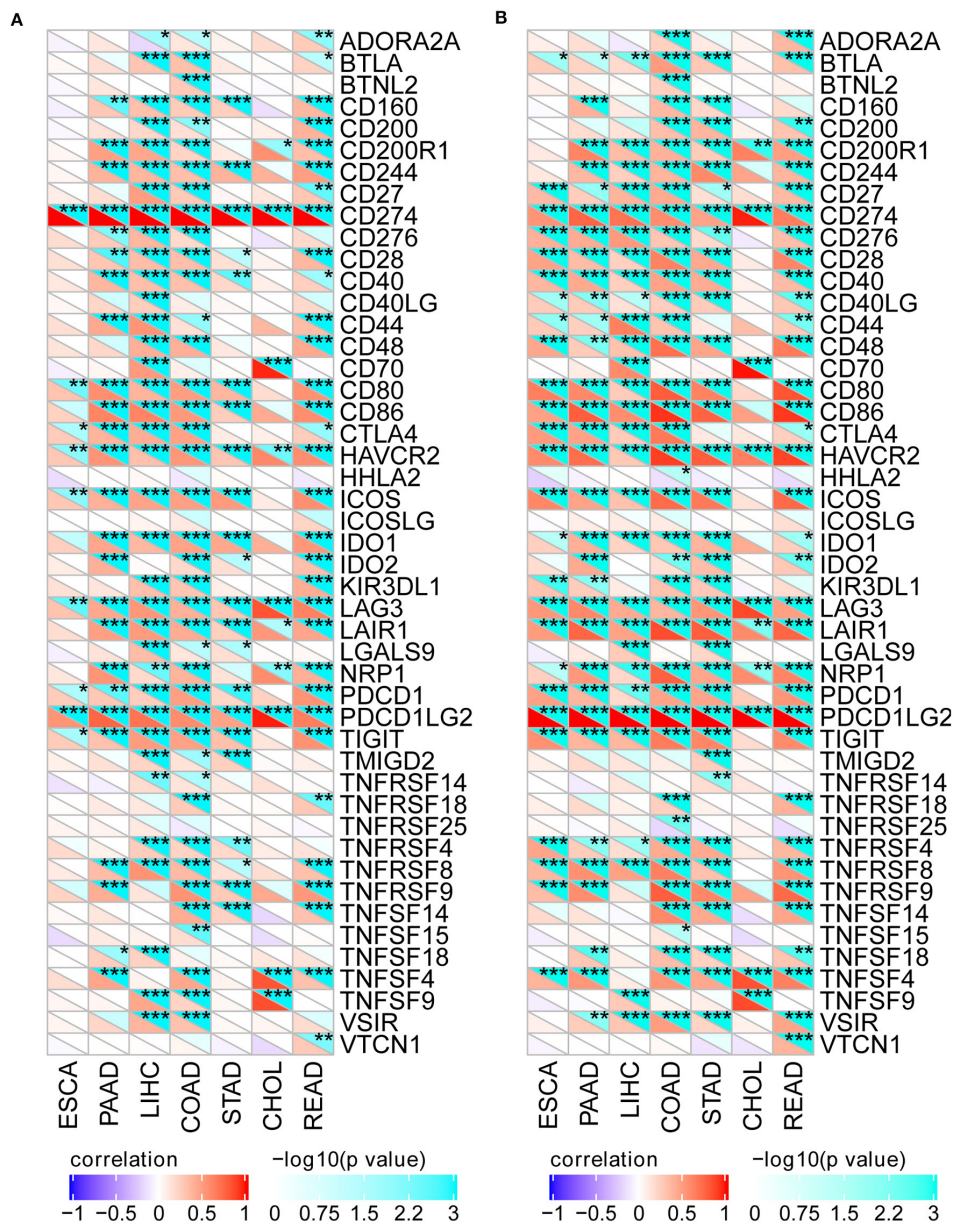
CD274/PDCD1LG2 expression is correlated with immune markers. **(A)** Heatmap shows that the CD274 expression is correlated with the tumor immune marker level across seven cancers. For each pair, right top triangle is colored representing a *p*-value; left bottom is colored representing a correlation coefficient. **(B)** Heatmap shows that the PDCD1LG2 expression is correlated with the tumor immune marker level across seven cancers. *p < 0.05; **p < 0.01; ***p < 0.001.

To better investigate the association of CD274/PDCD1LG2 expression with different immune infiltrating cells, we analyzed the relationship of CD274/PDCD1LG2 expression with the gene markers in diverse immunocytes, as shown in Figure 4. Our results suggested that CD274/PDCD1LG2 expression was significantly correlated with many immune markers in diverse immunocytes and distinct T cells.

### Correlation Analysis on TMB, MSI, MMR, and DNMT

**Figure 5:**
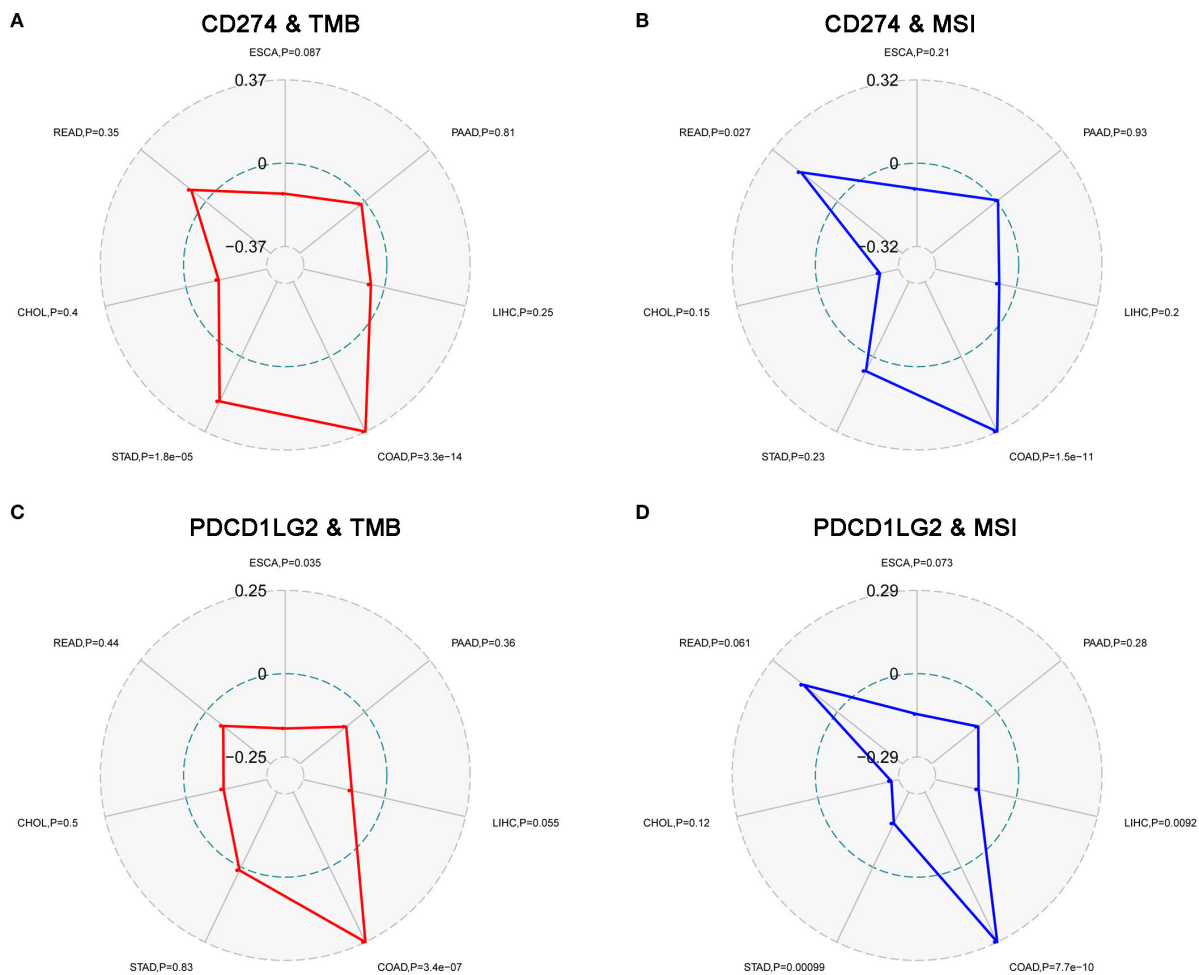
CD274/PDCD1LG2 expression is correlated with tumor mutation burden (TMB) and microsatellite instability (MSI). **(A)** Radar chart displaying a correlation between CD274 and TMB across seven cancers. Red line representing the Spearman correlation coefficient. **(B)** Radar chart displaying a correlation between CD274 and MSI. Blue line representing the Spearman correlation coefficient. **(C)** PDCD1LG2 expression and TMB. **(D)** PDCD1LG2 and MSI.

Moreover, we evaluated the association of TMB/MSI with CD274/PDCD1LG2 expression as shown in Figure 5. We discovered that the CD274 expression was correlated with the TMB in STAD (*p* = 1.8e-5), and COAD (*p* = 3.3e-14); while PDCD1LG2 expression was correlated with the TMB in ESCA (*p* = 0.035), and COAD (*p* = 3.4e-7), as presented in Figures 5A,C. Moreover, CD274 expression was found to be related to the MSI in COAD (*p* = 1.5e-11), and READ (*p* = 0.027); whereas PDCD1LG2 expression was associated with the MSI in COAD (*p* = 7.7e-10), LIHC (*p* = 0.009), and STAD (*p* = 0.001), as displayed in Figures 5B,D.

**Figure 6:**
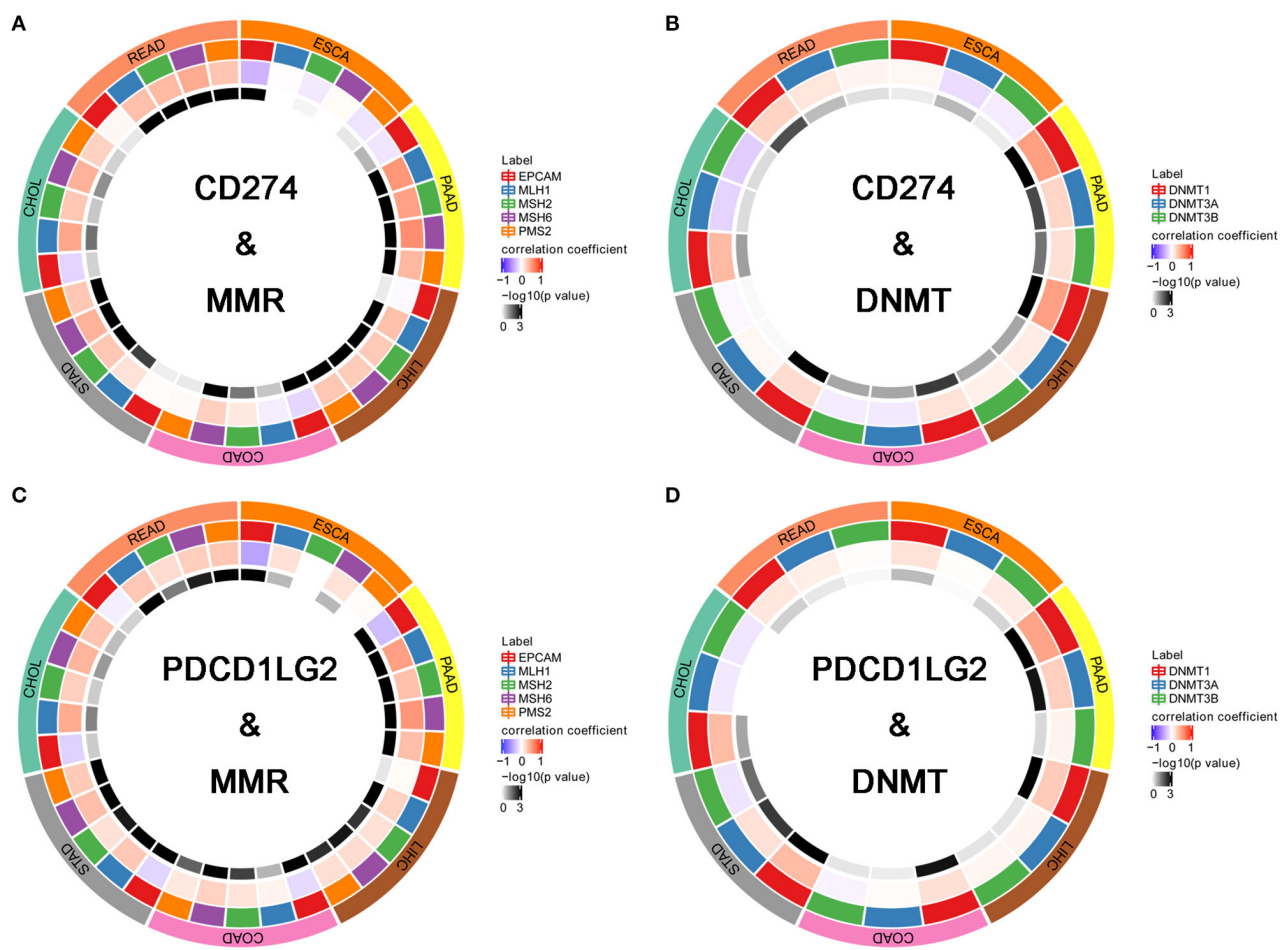
CD274/PDCD1LG2 expression is correlated with mismatch repair (MMR) and DNA methyltransferase (DNMT). **(A)** Circle chart displaying the overlap between CD274 and MMR across seven cancers. The first outer ring representing cancer types; the second outer ring representing five MMR genes; the third outer ring representing a correlation coefficient; and the fourth outer ring is colored representing a *p*-value. **(B)** CD274 and DNMTs. **(C)** PDCD1LG2 and MMRs. **(D)** PDCD1LG2 and DNMTs.

Further, we performed a correlation analysis between MMR genes (MLH1, MSH2, MSH6, PMS2, and EPCAM) and CD274/PDCD1LG2 as shown in Figures 6A,C. In all seven cancer types, CD274/PDCD1LG2 was correlated with at least one MMR gene. Besides, we also conducted a correlation analysis between DNMT (DNMT1, DNMT2, DNMT3A, and DNMT3B) and CD274/PDCD1LG2. As a result, in all seven cancer types, CD274/PDCD1LG2 was correlated with at least one DNMT gene (Figures 6B,D). Detailed correlation results are summarized in Table 1.

**Table 1:** Correlation analysis of CD274/PDCD1LG2 expression with mismatch repair (MMR) and DNA methyltransferase (DNMT).

	**Tumor**	**Spearman**	**MMR**					**DNMT**		
			**EPCAM**	**MLH1**	**MSH2**	**MSH6**	**PMS2**	**DNMT1**	**DNMT3A**	**DNMT3B**
CD274	CHOL	*R*	−0.18816	0.453539	0.289318	0.400772	0.235521	0.359917	−0.18746	−0.21202
	CHOL	*P*-value	0.333454	0.027339	0.260987	0.061694	0.333454	0.093221	0.4289	0.4289
	COAD	*R*	−0.17445	−0.07269	0.119258	0.249567	0.024473	0.144956	−0.0889	−0.08137
	COAD	*P*-value	0.000702	0.240673	0.031913	3.11E-07	0.601398	0.00561	0.114561	0.114561
	ESCA	*R*	−0.32706	0.009353	−0.08905	0.026681	−0.11629	0.041869	−0.148	−0.07887
	ESCA	*P*-value	0.000108	1	0.779336	1	0.56227	0.636896	0.180519	0.636896
	LIHC	*R*	−0.02565	0.323872	0.276122	0.268484	0.287001	0.490804	0.09953	0.08108
	LIHC	*P*-value	0.621419	7.39E-10	1.78E-07	2.81E-07	6.67E-08	1.57E-23	0.109575	0.117999
	PAAD	*R*	−0.10438	0.533641	0.48648	0.576787	0.36405	0.496813	0.213342	0.163804
	PAAD	*P*-value	0.165564	6.89E-14	1.74E-11	1.79E-16	1.17E-06	5.24E-12	0.008492	0.028903
	READ	*R*	0.042965	0.329019	0.355855	0.428334	0.300045	0.225528	0.128959	0.056493
	READ	*P*-value	0.581419	4.25E-05	9.46E-06	3.86E-08	0.000163	0.010155	0.193449	0.468364
	STAD	*R*	0.020888	0.151739	0.287854	0.392907	0.293081	0.183127	0.040141	−0.03326
	STAD	P-value	0.686804	0.006445	4.13E-08	1.36E-14	2.92E-08	0.001093	0.876637	0.876637
PDCD1LG2	CHOL	*R*	−0.21905	0.430888	0.248649	0.385328	0.306821	0.351351	−0.09472	−0.10579
	CHOL	*P*-value	0.287307	0.043513	0.287307	0.08119	0.206213	0.106864	1	1
	COAD	*R*	−0.17776	0.065562	0.142451	0.236716	0.122061	0.161801	0.021428	−0.05155
	COAD	*P*-value	0.000525	0.161284	0.006733	1.49E-06	0.017852	0.001526	0.647399	0.541866
	ESCA	*R*	−0.44693	0.157969	0.000631	0.148759	0.020448	0.146261	0.01071	0.105305
	ESCA	*P*-value	1.25E-08	0.17871	1	0.17871	1	0.189854	0.892398	0.36464
	LIHC	*R*	0.030398	0.231375	0.157928	0.181724	0.16655	0.273517	0.057927	0.053573
	LIHC	*P*-value	0.558383	3.17E-05	0.00444	0.001679	0.003735	2.4E-07	0.528893	0.528893
	PAAD	*R*	−0.32388	0.493875	0.324009	0.537676	0.331045	0.472252	0.249401	0.063617
	PAAD	*P*-value	2.05E-05	9.87E-12	2.05E-05	5.02E-14	1.91E-05	8.5E-11	0.001575	0.398879
	READ	*R*	−0.07558	0.287624	0.182248	0.258266	0.294101	0.125439	0.082768	0.01599
	READ	*P*-value	0.331687	0.000656	0.036821	0.002257	0.000572	0.318773	0.575208	0.837492
	STAD	*R*	−0.20015	0.279894	0.159715	0.333779	0.277346	0.350788	0.15516	−0.11656
	STAD	*P*-value	0.00019	1.41E-07	0.001919	1.64E-10	1.43E-07	8.03E-12	0.005175	0.023985

## Discussion

The present work illustrated a comprehensive workflow for a pan-cancer analysis and thoroughly investigated the role of CD274/PDCD1LG2 in gastrointestinal cancers. The CD274/PDCD1LG2 expression among the different cancer cell lines was reported. It was found that most cancer types showed a CD274/PDCD1LG2 alteration frequency, and the abnormal expression served as a prognostic factor in some cancer types. Serum CD274/PDCD1LG2 levels could predict disease-free survival after the CIK cell therapy in patients with LIHC. More importantly, the CD274/PDCD1LG2 expression was associated with the cancer immunity. Furthermore, CD274/PDCD1LG2 was identified to be correlated with TMB, MSI, MMR, and DNMT.

It is significant to identify the abnormal gene expression among the different types of cancers. Also, it is of great significance to identify the tumor-specific targets or tumor-related features in individualized treatment, thus enhancing the possibility of curing among the patients with cancer (21). A pan-cancer analysis of CD274/PDCD1LG2 is valuable for identifying a differential expression and its role in many cancer types (22, 23). Using CCLE and TCGA databases, a large number of diverse types of cancers are obtained, which contributes to discovering the abnormal CD274/PDCD1LG2 expression among the different types of cancers. Besides, a thorough pan-cancer cellular analysis of gene expression can be performed through CCLE, which sheds light on the future cellular experiments. On the other hand, TCGA genomic and survival analyses may provide guidance for clinical practice and future studies.

In recent years, immunotherapy has achieved prominent efficacy in the treatment of tumors. Notably, the present work also demonstrated that the expression of CD274/PDCD1LG2 was related to cancer immunity. According to this study, the CD274/PDCD1LG2 level was related to the immune infiltration levels in cancers. ESTIMATE has been reported as a metric for the evaluation of the prognosis for the patients with cancer (24). Numerous recent studies have employed the ESTIMATE method to assess various tumors, and it has been successfully applied to the genome data. For instance, ESTIMATE is used to predict the outcomes among the patients with glioblastoma and cutaneous melanoma (25, 26). Using a TCGA cohort, the ESTIMATE method was utilized to generate the immune and stromal scores. As a result, CD274/PDCD1LG2 was correlated with the ESTIMATE scores. Furthermore, CD274/PDCD1LG2 was also detected to be correlated with the gene markers in infiltrating cells as observed from Figure 4.

Gene mutation is a major cause of cancer formation (27). Typically, mutations in some specific genes may predict the patient prognosis and treatment response (28, 29). An adaptive immune system can identify and detect cancers through the non-self neoantigens associated with somatic mutations. Therefore, TMB affects the possibility of generation of an immunogenic peptide, thus impacting the patient response to immune checkpoint inhibitors (30, 31). Consequently, it is of great significance to comprehensively investigate the association of the CD274/PDCD1LG2 expression with TMB levels among the patients with cancer, using the TCGA-derived high-quality matched data. Moreover, TMB and MSI also indicate the production of new antibodies. Noteworthily, a number of cases with high MSI (MSI-H) show increased TMB levels (32). Further, as discovered by Bonneville et al. (33), the MSI-H cervical squamous cell carcinoma and adrenocortical carcinoma showed abnormally high mutation frequencies. On the other hand, MSI is a vital index to predict the tumor genesis and development (19). The National Comprehensive Cancer Network (NCCN) guidelines have recommended an MSI testing for all READ subtypes, and the READ mortality can be reduced by the early detection of MSI (34). Some studies have shown that the MMR defect (dMMR) in cancer cells produces antigens that can be easily recognized by T cells, in this way, the PD-1 inhibitors are highly effective on the MSI-H solid tumors (35). In this regard, FDA has approved the use of Keytruda for the treatment of MSI-H/dMMR solid tumors (36). Therefore, TMB, MSI, and MMR can serve as a predicting factor for the efficacy of immunotherapy at present. We found in this study that the CD274/PDCD1LG2 expression was correlated with TMB and MSI in some cancer types. Moreover, the gene DNA methylation can regulate gene expression, while such a change can be utilized by tumor cells to destroy the immunogenicity and the immune recognition mechanisms, thus obtaining the immune escape phenotypes. Notably, the application of a methylase inhibitor in combination with an immunocheckpoint inhibitor has achieved initial advantages (20). An analysis between CD274/PDCD1LG2 and a methylase inhibitor is expected to inject a new vitality into the cancer treatment. Taken together, our findings provide clues for the association between CD274/PDCD1LG2 and cancer immunity, which require further experiment investigations to clarify.

Collectively, our comprehensive pan-cancer analysis has characterized CD274/PDCD1LG2 in different cancer cell lines and tissues of seven gastrointestinal cancer types. Our new integrative omics-based workflow might provide novel insights into the patients with gastrointestinal cancer regarding immunotherapy.

## Data Availability Statement

The datasets presented in this study can be found in online repositories. The names of the repository/repositories and accession number(s) can be found in the article/Supplementary Material.

## Author Contributions

WL conceived of and designed the study, supervised the study, and reviewed the manuscript. LD and ZH performed the literature search, collected and analyzed the data, generated the figures and tables, and wrote the manuscript. All authors contributed to the article and approved the submitted version.

## Conflict of Interest

The authors declare that the research was conducted in the absence of any commercial or financial relationships that could be construed as a potential conflict of interest.

## Supplementary Material

Supplementary Figure 1 CD274/PDCD1LG2 expression and its correlation with colon adenocarcinoma (COAD) patient survival.

Supplementary Figure 2 CD274/PDCD1LG2 expression and its correlation with esophageal carcinoma (ESCA) patient survival.

Supplementary Figure 3 CD274/PDCD1LG2 expression and its correlation with liver hepatocellular carcinoma (LIHC) patient survival.

Supplementary Figure 4 CD274/PDCD1LG2 expression and its correlation with pancreatic adenocarcinoma (PAAD) patient survival.

Supplementary Figure 5 CD274/PDCD1LG2 expression and its correlation with rectum adenocarcinoma (READ) patient survival.

Supplementary Figure 6 CD274/PDCD1LG2 expression and its correlation with stomach adenocarcinoma (STAD) patient survival.

Supplementary Figure 7 Serum CD274/PDCD1LG2 level and its correlation with disease free survival in LIHC CIK patients. **(A)** Serum CD274 (PDL1) distribution and cutoff of PDL1 level. **(B)** Lower serum PDL1 level was associated with longer disease free survival. **(C,D)** PDCD1LG2 (PDL2).

## Abbreviations

PD-1: programmed death 1
TCGA: The Cancer Genome Atlas
CCLE: Cancer Cell Line Encyclopedia
CIK: cytokine-induced killer
ESTIMATE: MAlignant Tumor tissues using the Expression data
TMB: tumor mutation burden
MSI: microsatellite instability
MMR: mismatch repair
DNMT: DNA methyltransferase
GTEx: Genotype-Tissue Expression
ROC: Receiver operating characteristic
KM: Kaplan–Meier
OS: overall survival
CHOL: cholangiocarcinoma
COAD: colon adenocarcinoma
ESCA: esophageal carcinoma
LIHC: liver hepatocellular carcinoma
PAAD: pancreatic adenocarcinoma
READ: rectum adenocarcinoma
STAD: stomach adenocarcinoma.
