# Supplementary figures and images for: Analysis of the PD-1 Ligands Among Gastrointestinal Cancer Patients: Focus on Cancer Immunity
Source: Front Oncol. 2021 Mar 23;11:637015. doi: 10.3389/fonc.2021.637015 (PMC8021907; doi:10.3389/fonc.2021.637015)

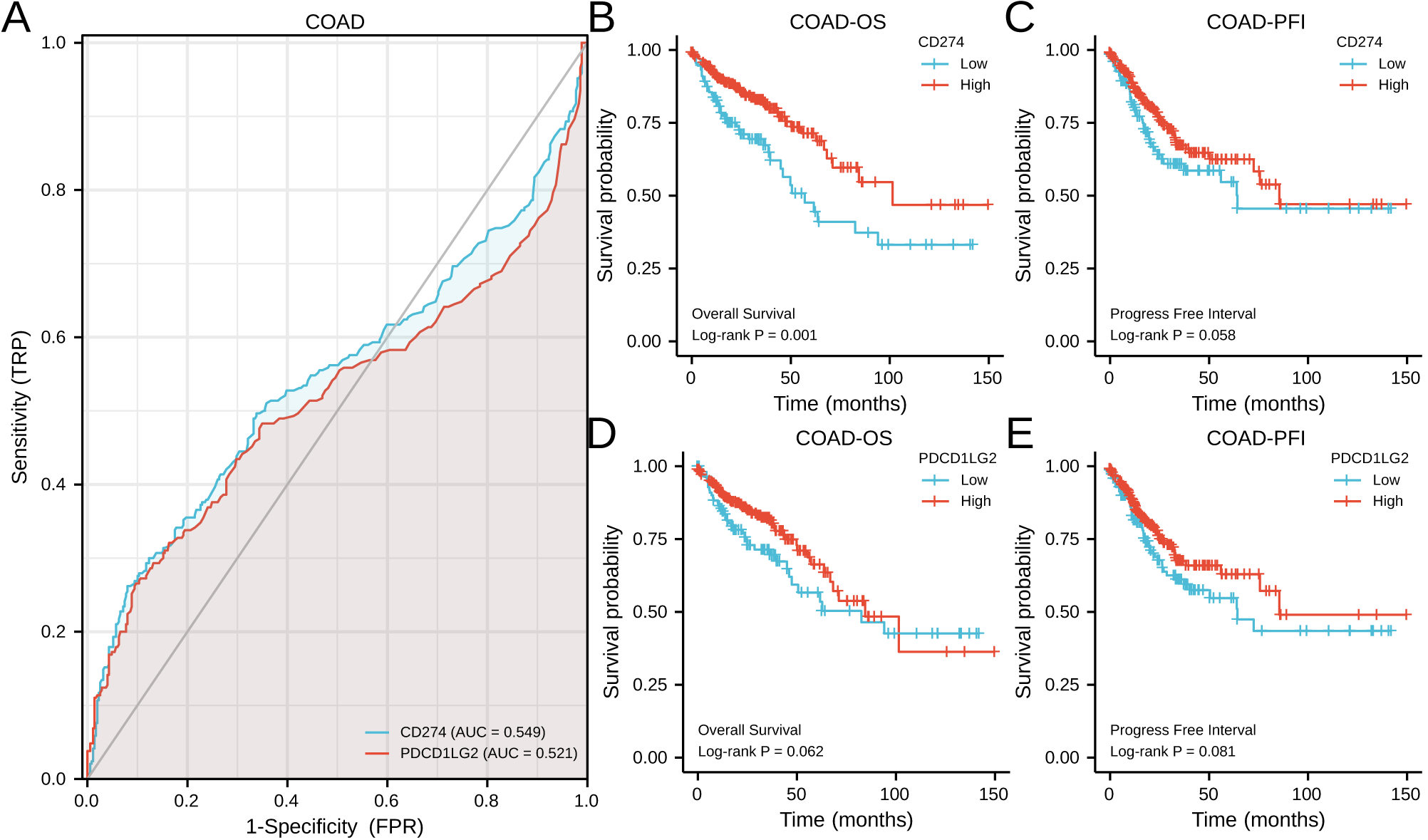

Supplement: Supplementary Figure 1 — CD274/PDCD1LG2 expression and its correlation with colon adenocarcinoma (COAD) patient survival. [file Image_1.TIF]

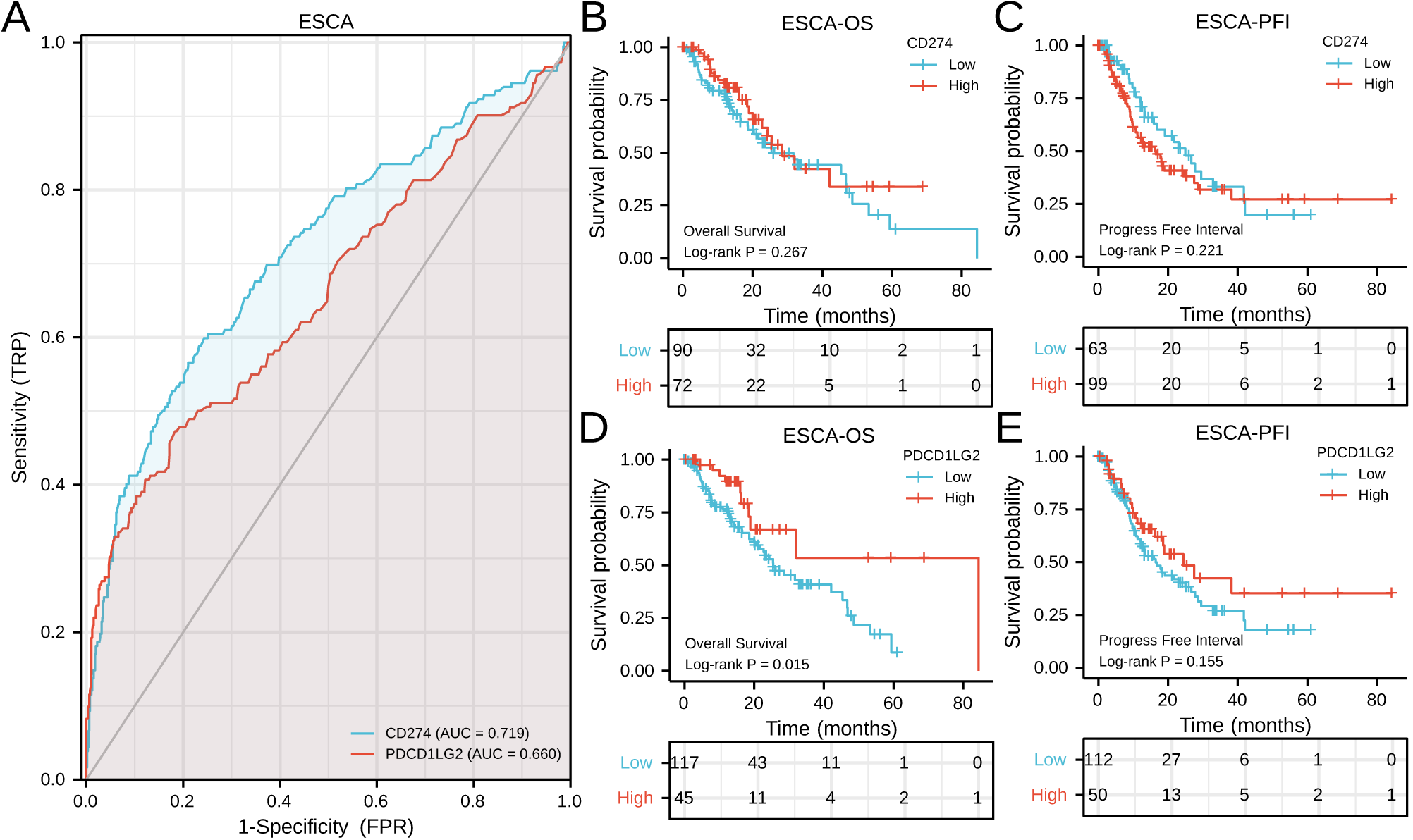

Supplement: Supplementary Figure 2 — CD274/PDCD1LG2 expression and its correlation with esophageal carcinoma (ESCA) patient survival. [file Image_2.TIF]

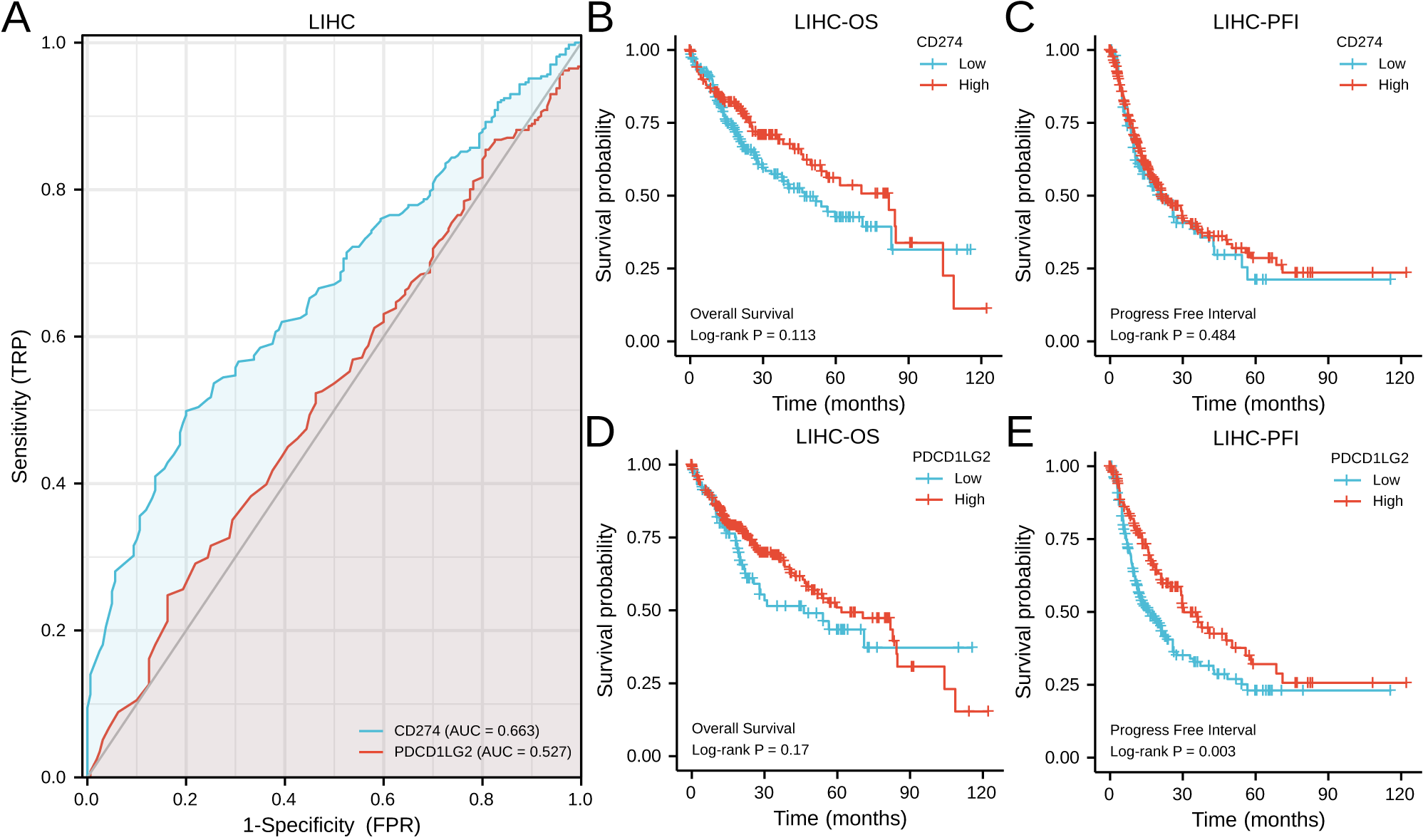

Supplement: Supplementary Figure 3 — CD274/PDCD1LG2 expression and its correlation with liver hepatocellular carcinoma (LIHC) patient survival. [file Image_3.TIF]

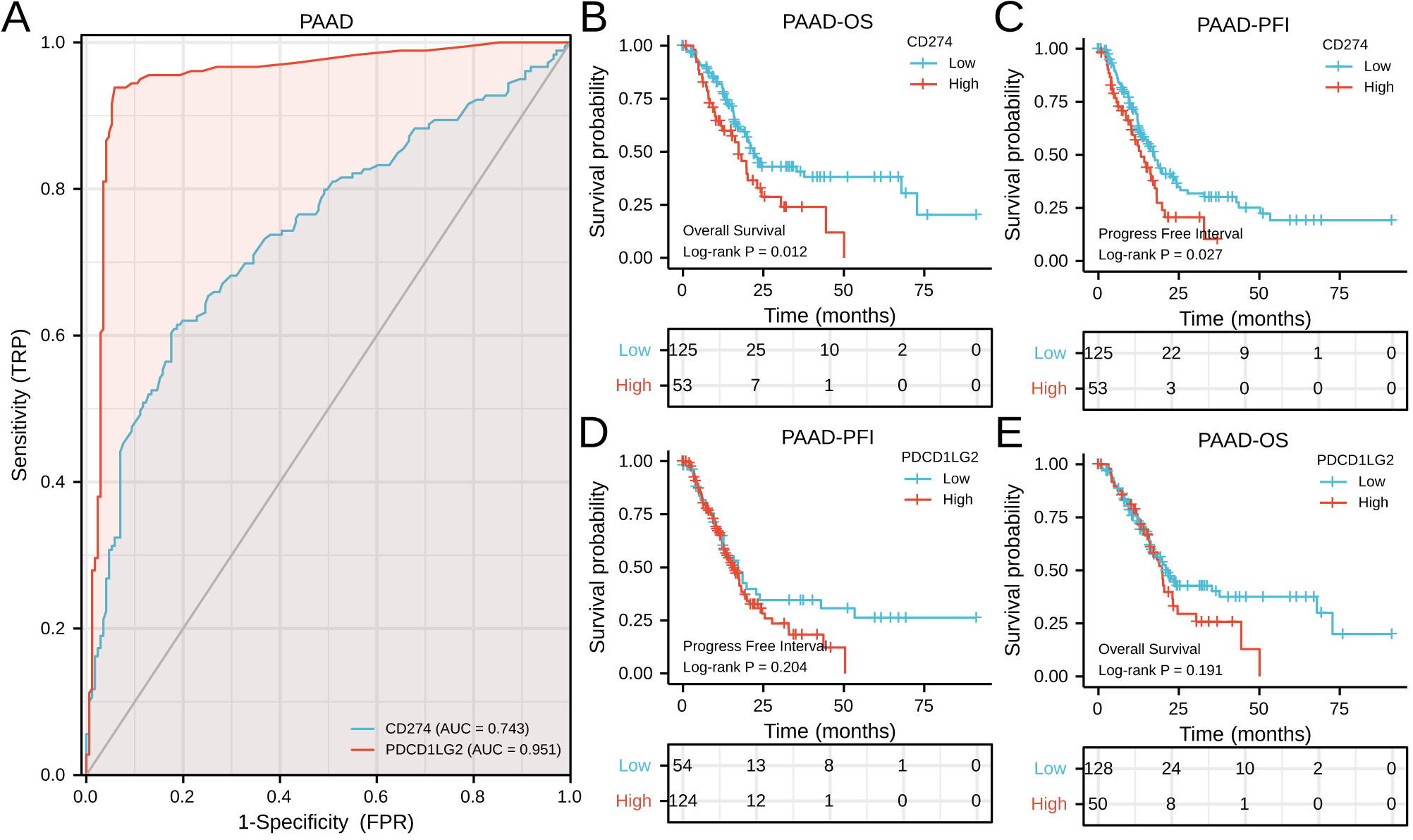

Supplement: Supplementary Figure 4 — CD274/PDCD1LG2 expression and its correlation with pancreatic adenocarcinoma (PAAD) patient survival. [file Image_4.TIF]

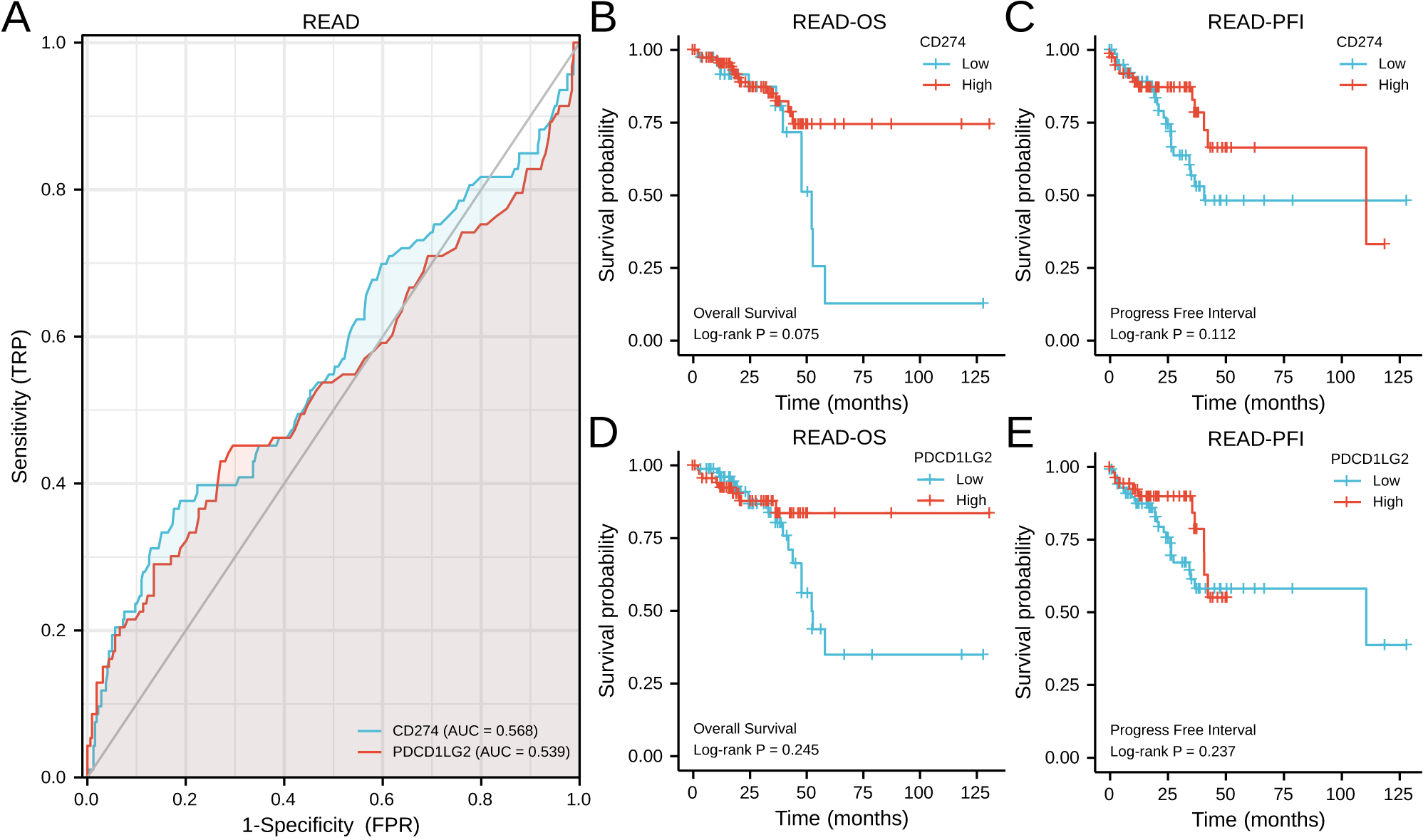

Supplement: Supplementary Figure 5 — CD274/PDCD1LG2 expression and its correlation with rectum adenocarcinoma (READ) patient survival. [file Image_5.TIF]

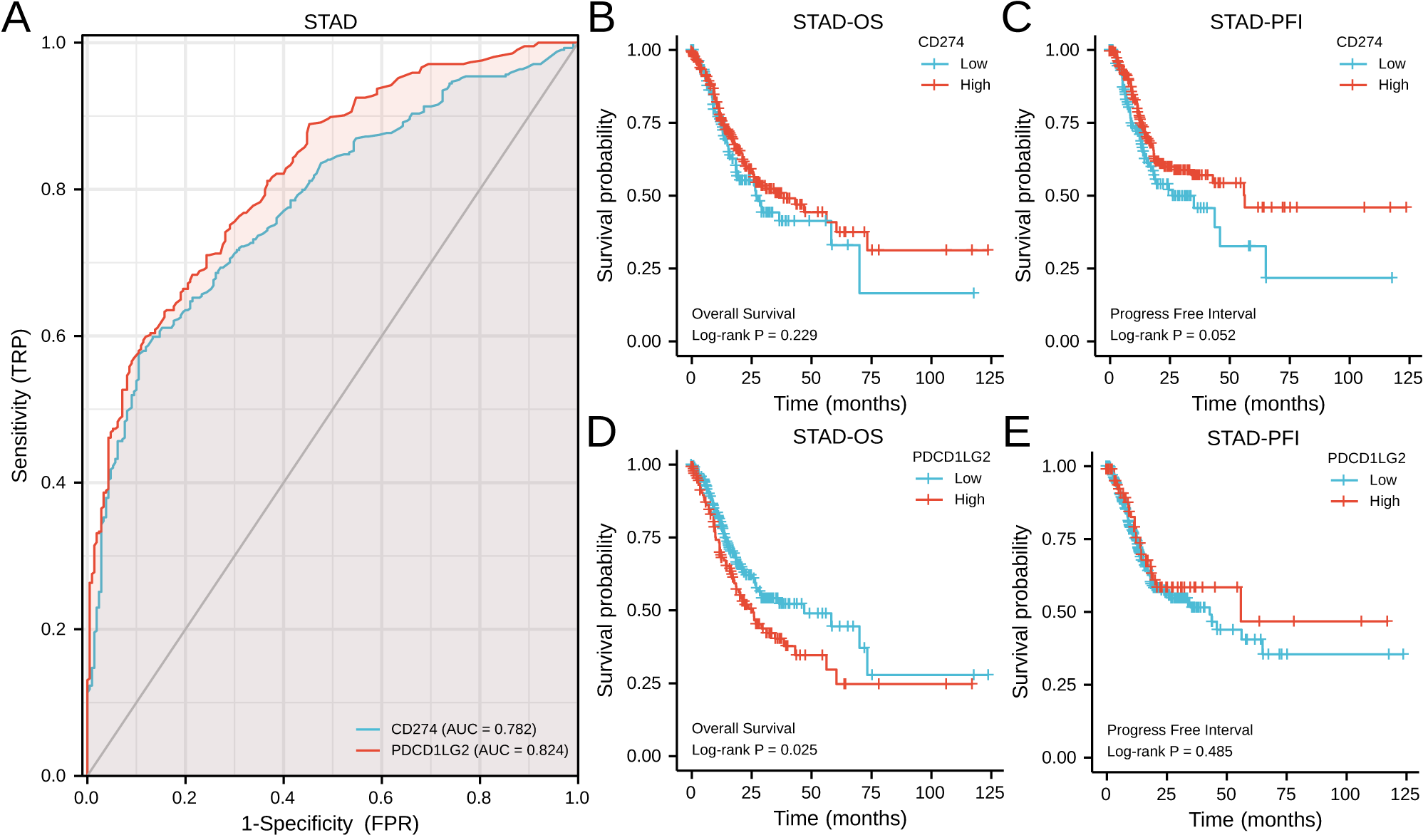

Supplement: Supplementary Figure 6 — CD274/PDCD1LG2 expression and its correlation with stomach adenocarcinoma (STAD) patient survival. [file Image_6.TIF]

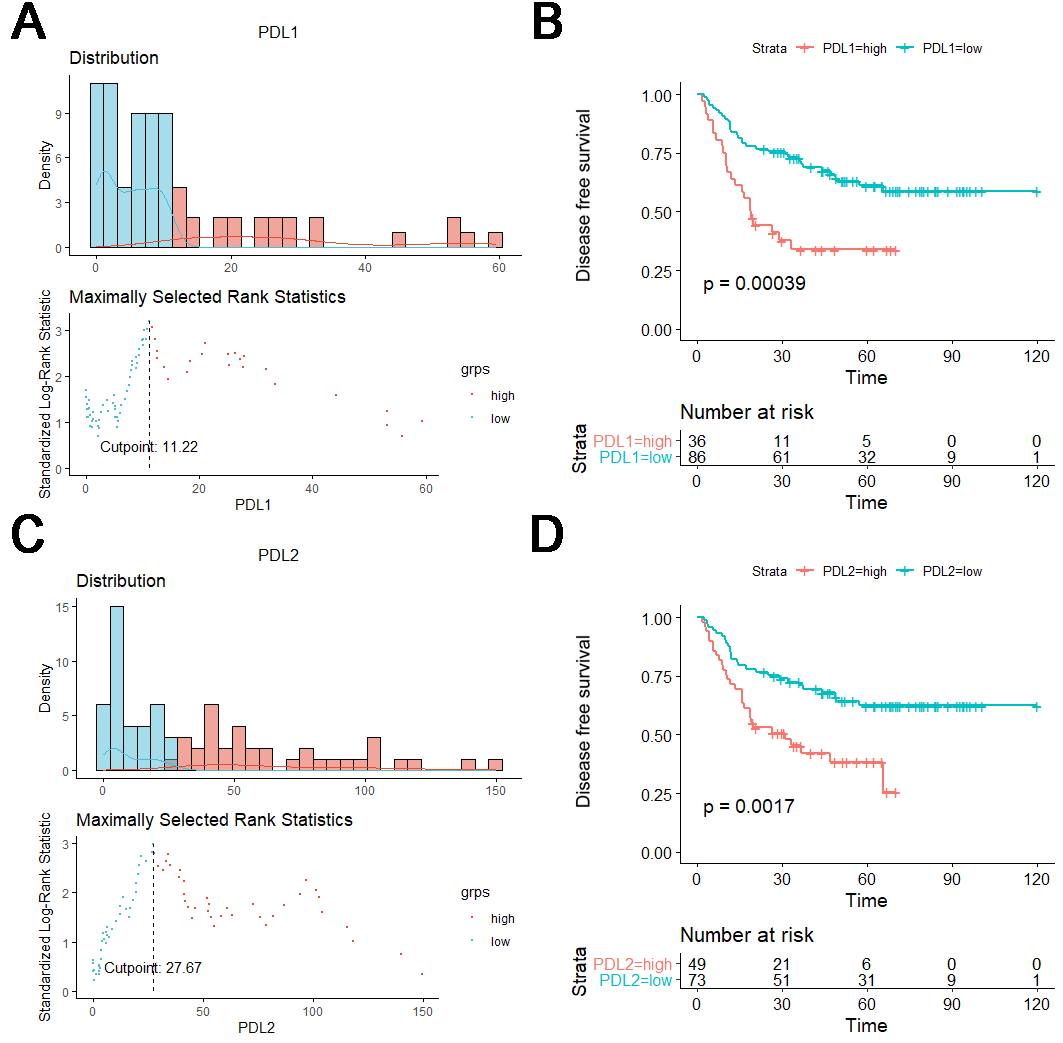

Supplement: Supplementary Figure 7 — Serum CD274/PDCD1LG2 level and its correlation with disease free survival in LIHC CIK patients. (A) Serum CD274 (PDL1) distribution and cutoff of PDL1 level. (B) Lower serum PDL1 level was associated with longer disease free survival. (C,D) PDCD1LG2 (PDL2). [file Image_7.TIF]
